# Surgical Treatment of a Severe Electrolyte Imbalance: A Case Report of an Elderly Patient With McKittrick–Wheelock Syndrome

**DOI:** 10.1155/cris/9986008

**Published:** 2025-10-10

**Authors:** Piotr Roman Więckowski, Joanna Matylda Łysak, Jakub Blicharz, Maciej Dudziński, Piotr Sienkiewicz

**Affiliations:** Department of Surgery and Vascular Surgery, John Paul II's Mazovian Provincial Hospital in Siedlce, Siedlce, Mazowieckie, Poland

## Abstract

McKittrick–Wheelock is a rare syndrome characterized by a severe, difficult to correct electrolyte imbalance, chronic mucus diarrhea, and a large rectal polyp. In this case report, we describe an elderly patient diagnosed with a large nonmalignant rectal polyp during a routine colonoscopy 10 years prior to admission. For years, the patient has suffered from diarrhea, causing episodic life-threatening hypokalemia and hyponatremia with several unsuccessful attempts at endoscopic polyp removal. Due to symptomatic cholelithiasis, the patient was transferred to the surgical ward and diagnosed with McKittrick–Wheelock syndrome. The patient had undergone cholecystectomy and, after a period of preoperative preparation, underwent an abdominoperineal resection of the rectum. Histopathologic evaluation revealed a low-grade (G1) rectal carcinoma. This case report highlights the importance of a careful assessment of patients with electrolyte level disturbances, with some, albeit very rarely, requiring surgical intervention.

## 1. Introduction

Hypokalemia and hyponatremia can cause severe, life-threatening complications. Low serum potassium levels may result in arrhythmias, while hyponatremia can lead to cerebral swelling and cerebral herniation. The root cause of those electrolyte disturbances is varied [1]. Mucoid secretions can have a high electrolyte content, resulting in a profound loss of essential ions. This is the case in the McKittrick–Wheelock syndrome, which is characterized by chronic mucous diarrhea, acute kidney injury (AKI) with an electrolyte disbalance and a large rectal tumor [2]. The tumor is usually identified as a rectal adenoma; however, as much as 40% of the polyps larger than 2 cm show signs of malignancy [3]. This condition requires surgery, as episodes of severe electrolyte loss carry a high risk of death [2]. The patient, whom we describe in this case report, has been misdiagnosed for 10 years and was hospitalized multiple times during this period in different internal medicine and neurology wards. The diagnoses this patient received were based on the complications caused by McKittrick–Wheelock syndrome, such as epilepsy, related to the extremely low serum sodium concentration and various rare renal and endocrine disorders, serving as an explanation for bouts of extreme ion depletion. There was no connection established between a large rectal polyp and episodes of AKI and extreme ion loss.

## 2. Case Presentation

A female patient in the eighth decade of her life was hospitalized in the Internal Medicine ward due to a severe electrolyte disbalance and dehydration resulting in an AKI with potassium levels at 2.88 mmol/L. In addition to a severe life-threatening electrolyte disbalance the patient exhibited symptoms of cholelithiasis.

**Figure 1:**
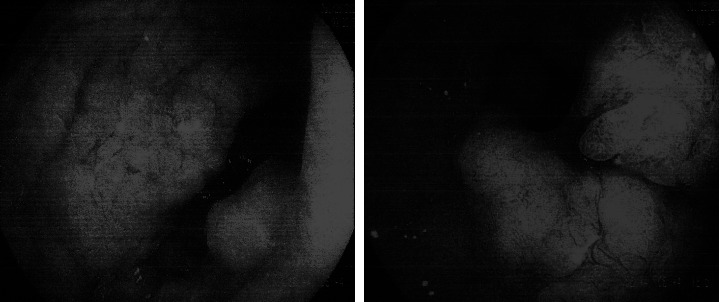
A large villous rectal polyp encompassing most of the rectal mucosa circumference. The tumor was described as benign by three different endoscopists at different hospitals.

The patient's history is noteworthy for repeated Emergency Ward and Internal Medicine Ward admissions due to severe dehydration combined with extreme hyponatremia (lowest level being 115 mmol/L) and hypokalemia (lowest level being 2.63 mmol/L). She had undergone numerous radiologic examinations due to a large polyp located in the rectum, first diagnosed 10 years before her current admission. A colonoscopy had been performed with the diagnosis of a benign villous polyp of the rectum. The polyp was described as encompassing more than two-thirds of the rectal circumference and roughly 7 cm in length.  Figure 1.

**Figure 2:**
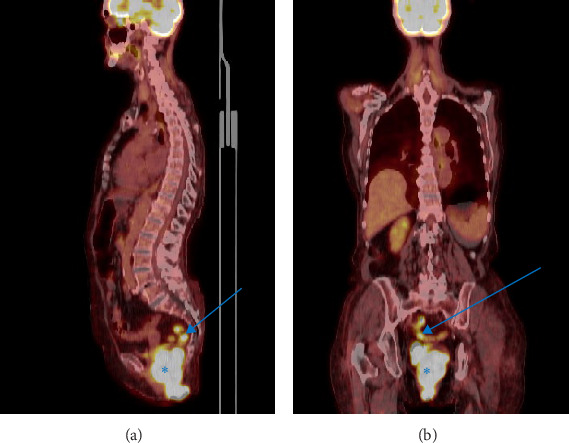
A PET scan superimposed on a CT scan. Image A –a sagittal view. Image B– a coronal view. Blue arrow points to the sigmoid colon with a mildly elevated glucose metabolism −11.56 g/mL standardized uptake value (SUV). Blue “*⁣*^*∗*^” symbol marks the rectal tumor with an elevated glucose metabolism −23.58 g/mL SUV. The elevated glucose metabolism was attributed to the inflammatory process, with no suspicion of malignancy raised. No lymph nodes with increased glucose metabolism were found.

Endoscopic removal undertaken a year prior to the admission had to be aborted due to massive bleeding during an attempt to remove a part of the polyp. A positron emission tomography (PET) scan performed 8 years prior to the current admission revealed increased 18-F-deoxyglucose metabolism in the polyp, which was attributed to the inflammatory process.  Figure 2.

The patient has a history of epileptic seizures exacerbated by electrolyte disturbances treated with daily carbamazepine, diagnosed 8 years prior to the current admission. The patient reported a mucous-like rectal discharge that occurs continuously due to incontinence, with the discharge volume approaching several liters per day. Due to the constellation of symptoms (several episodes of an AKI due to dehydration, hyponatremia, hypokalemia, and a large villous rectal polyp), a suspicion of McKittrick–Wheelock syndrome was raised.

Due to two separate surgical problems—the rectal polyp and cholelithiasis—combined with multiple comorbidities, a decision was made to pursue the treatment of cholelithiasis first.

The laparoscopic cholecystectomy was uneventful with a relatively fast recovery. The presence of difficult to correct hypokalemia and hyponatremia was noted both pre- and postoperatively.

The patient had been admitted 1 week before the second surgery in order to achieve correction of hypokalemia and hyponatremia alongside nutritional treatment.

The preoperative course was complicated with several bouts of extreme hypokalemia with potassium levels below 3 mmol/L despite treatment with both oral and intravenous potassium. A central line was established to administer a more concentrated potassium chloride solution. Nutritional treatment was delivered both orally using oral nutritional solutions and intravenously.

The abdominoperineal resection was performed in two stages. In the first stage, the rectum, along with the mesorectum, was resected laparoscopically. The resection was performed in the avascular space. The superior rectal artery and vein were found, dissected, and ligated using metal clips, sparing the main trunk of the inferior mesenteric artery. The second, perineal, stage of the operation commenced. The skin around the anus was cut, and the rectum was resected with the mesorectum en bloc. The rectum was resected with the mesorectum en bloc, while avoiding injury to the pelvic floor muscles. Extensive irrigation of the empty post-resection space was used. The pelvic floor was closed in layers. Then, the pneuomoperitoneum was reestablished, and the stoma opening in the left hypogastric space was made around the trocar. The sigmoid colon stump was used to establish the final colostomy.

Postoperatively, the patient's status was stable; however, due to hypotension, she required an infusion of low-dose noradrenaline (0.5 mg/h). The cessation of potassium supplementation occurred on the second day postsurgery. The operation was complicated by the postoperative paralytic ileus, which lasted for 7 days. At the end of the patient's hospital stay, the electrolyte levels were normal and stable, urine output was satisfactory, and creatinine levels were normal, adjusted for the patient's age. The resolution of diarrhea occurred immediately post-surgery, with stoma outputs being of standard volume after the resolution of a postoperative ileus. The patient's condition improved dramatically with the return to daily activities, which the patient had abandoned due to chronic diarrhea. Despite the patient's age, she was able to walk long distances using a walker, perform all personal hygiene tasks, and properly care for a stoma. The patient's mental state improved. The resolution of epileptic seizures was achieved, which we attribute mainly to normalization of electrolyte levels. The patient was referred to the neurologist with the intention of weaning the patient off carbamazepine completely.

The excised specimen measured 17 cm in length and contained a rectum with mesorectum fragments and an anus with adjacent skin. The macroscopic margin was 7 cm proximally and 2 cm distally. The exophytic tumor size was measured to be 14 cm and encompassed the whole circumference of the rectal mucosa.

**Figure 3:**
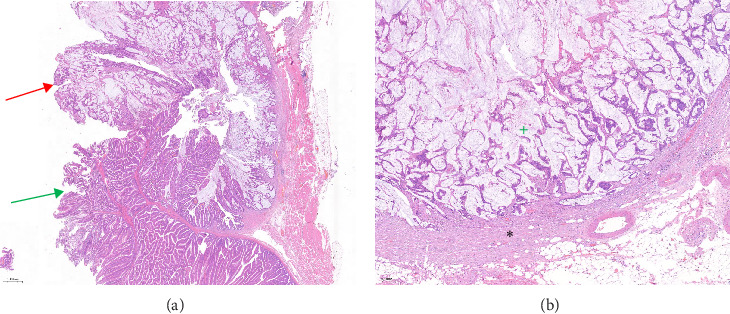
Histopathology slides. Image 3A shows the villous adenoma, marked with the green arrow, adjacent to the mucinous adenocarcinoma, marked with the red arrow. Mucinous adenocarcinoma is characterized by the presence of abundant extracellular mucin within the tumor. Image 3B shows the mucinous adenocarcinoma infiltrating the muscular layer of the colon. The green “+” symbol marks the extracellular mucin. The black “*⁣*^*∗*^” symbol marks the muscularis propria of the colon.

The histopathology report revealed a G1 (low-grade) mucinous rectal adenocarcinoma with a partially gelatinous structure adjacent to the villous adenoma.  Figure 3A. Most of the tumor did not cross the mucosa. Focal infiltration of the mucosa, submucosa, and muscular layer into the mesorectum was noted. The deepest infiltration occurred 5 cm from the distal margin with a 1 mm radial margin.  Figure 3B. No signs of either vascular or nerve infiltration were found. Eleven lymph nodes were found in the mesorectum, with none exhibiting signs of cancerous infiltration. A single lymph node near the vascular margin was also free of cancer cell infiltration.

After consultation with the hospital's oncology team, it was decided not to pursue further radiotherapy or chemotherapy due to the patient's age, comorbidities, and the tumor characteristics. With the negative surgical margin and no lymph node infiltration, it is safe to assume that the tumor will not recur during the patient's lifetime.

**Figure 4:**
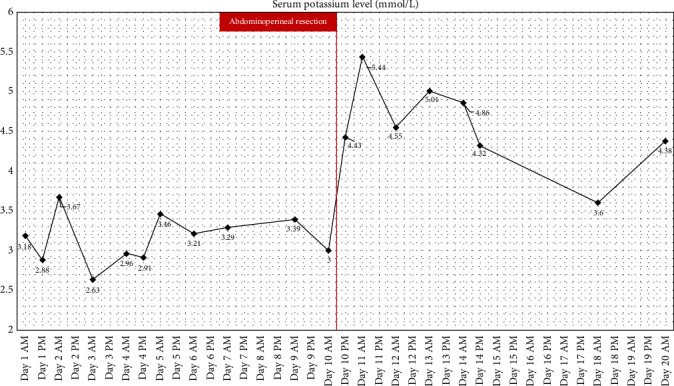
Serum potassium levels during the Patient's hospital stay. Red line shows the date of the abdominoperineal resection. A notable elevation of the serum potassium level can be seen after the surgery.

**Figure 5:**
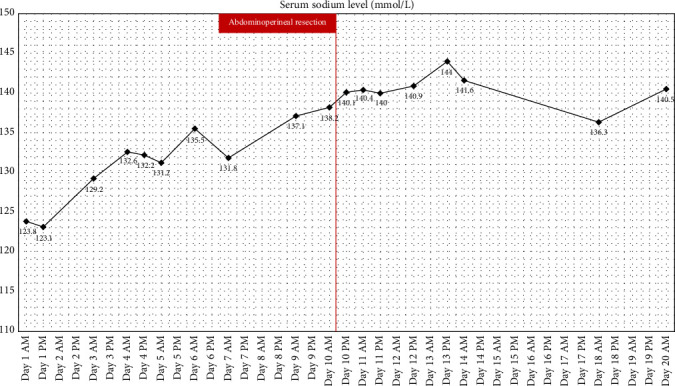
Serum sodium levels during the Patient's hospital stay. Red line shows the date of the abdominoperineal resection. A notable elevation and stabilization of the serum sodium level can be observed after the surgery.

In both the Figure 4 and the Figure 5 we provide the patient treatment course, with Figure 4 showing patient's potassium levels while Figure 5 shows patient's sodium levels in relation to time and treatments used. Supporting Information provides the serum electrolyte level data in table form. The electrolyte serum levels can be viewed in the table form in the Supporting Information spreadsheet.

## 3. Discussion

McKittrick–Wheelock syndrome is a rare cause of electrolyte disbalance in elderly patients. The rectal villous adenoma secretes a mucus high in electrolyte content, causing a profound ion loss which leads to major complications. It should be noted that the majority of the patients experience numerous hospitalizations due to ion depletion prior to the definitive diagnosis [2]. This is caused, in our opinion, by insufficient knowledge about this rare disease. That situation carries an extensive risk of death for the patient, as severe hypokalemia can lead to deadly arrhythmias [1]. The order of procedures chosen for this patient is crucial. Laparoscopic cholecystectomy was performed first due to the symptomatic nature of the gallstones combined with the inherent risk of cholecystitis and cholangitis. If not performed first, the occurrence of cholecystitis and cholangitis post-abdominoperineal resection would certainly be lethal, considering the patient's age and comorbidities. Considering the patient's poor nutritional state at admission due to the constant diarrhea, the decision to pursue intensive inpatient nutritional treatment was made to facilitate healing after the second surgery. The extensiveness of the resection and possible major complications, especially in an elderly patient, are concerns raised by Ochard et al. [2] in their systematic review, however, we believe that after adequate preoperative preparation with multidisciplinary approach a curative radical approach is appropriate, as in the group of patients with no intervention the mortality was a staggering 61.5%, while in patients undergoing surgical procedures, this number was 5.2%. We acknowledge that such an extensive procedure carries an exceptionally high risk for this patient; however, the next episode of extreme electrolyte depletion could very well be fatal. Considering the inherent risks of the abdominoperineal resection and comparing them to the high mortality of AKI and hypokalemia, in our opinion, the operative approach is favored by a large margin. Less invasive, compared to the abdominoperineal resection, options are available; however, due to the unfavorable size (>3 cm) [3, 4] of the tumor, those procedures were deemed inappropriate, which is consistent with other authors' experience while dealing with large tumors [5]. The patient had undergone a single attempt at endoscopic resection, which resulted in massive bleeding requiring a blood transfusion; thus, no further endoscopic procedures had been attempted. A transanal excision could be attempted at a specialist center; however, it carried a high risk of morbidity and recurrence [2]. We aimed to avoid the need for repeat procedures to avoid the morbidity associated with general anesthesia in a frail elderly patient. In the case of our patient, a sphincter-preserving surgery was not pursued due to the tumor location and the presence of incontinence prior to the surgery. Usually, McKittrick–Wheelock syndrome is described as being caused by a benign villous adenoma; however, cases of malignancy have been described. The work of Villanueva et al. [5] describes two patients with adenocarcinomas found in the resected polyp and includes a literature review of 10 further cases, which shows that malignancy occurs most commonly in tumors larger than 4.5 cm and in older patients (over the age of 55). This corresponds to our patient's characteristics. This report also shows that the initial biopsy, as in our case, may reveal only a benign adenoma despite the presence of adenocarcinoma infiltrates in the polyp [5]. Medical management using octreotide or indomethacin was not attempted due to inconclusive evidence of its effectiveness and a significant risk of drug interactions, especially with indomethacin. We managed to wean the patient off electrolyte supplementation completely and eliminate epileptic seizures completely. In addition, the psychological condition of the patient improved due to the resolution of incontinence, with stoma bag changes being more manageable and compatible with daily activities than frequent diaper changes. Despite an extreme electrolyte disbalance, poor preoperative nutritional status and advanced age of the patient, our treatment yielded excellent results. This result was made possible by the initiation of nutritional therapy, extensive physiotherapy, and vigilant care by nursing staff, along with a surgical intervention, which highlights the importance of multidisciplinary team care for patients with rare diseases.

## 4. Conclusions

McKittrick–Wheelock syndrome is a rare cause of life-threatening hypokalemia and hyponatremia with dehydration and AKI. It should be suspected in patients with large rectal tumors secreting mucus. A careful assessment of every patient presenting with such symptoms to the emergency ward, along with a treatment plan that extends beyond the usual electrolyte replacement therapy, should be indicated. Abdominoperineal resection, despite its inherent surgical risks and possible complications, remains the treatment of choice in patients with large tumors located low in the rectum.

## Data Availability

The data that supports the findings of this study are available in the supporting information of this article.

## Supporting Information

Supporting Information The Supporting Information consists of a Microsoft Excel spreadsheet containing serum sodium and serum potassium levels measured multiple times during the Patient's hospital stay. Accompanying graphs help visualize the elevation and stabilization of electrolyte levels after the abdominoperineal resection.

## References

[1] Laing C. (2011). Clinical Approach to Electrolyte Abnormalities. *Medicine*.

[2] Orchard M. R., Hooper J., Wright J. A., McCarthy K. (2018). A Systematic Review of McKittrick–Wheelock Syndrome. *The Annals of The Royal College of Surgeons of England*.

[3] Rutter M. D., Chattree A., Barbour J. A. (2015). British Society of Gastroenterology/Association of Coloproctologists of Great Britain and Ireland Guidelines for the Management of Large Non-Pedunculated Colorectal Polyps. *Gut*.

[4] Cowan M., Silviera M. (2016). Management of Rectal Polyps. *Clinics in Colon and Rectal Surgery*.

[5] Villanueva M. E. P., Onglao M. A. S., Tampo M. M. T., Lopez M. P. J. (2022). McKittrick-Wheelock Syndrome: A Case Series. *Annals of Coloproctology*.

